# Kinesiological Treatment of Early Spine Osteoarthritis in a Motorcyclist

**DOI:** 10.3390/ijerph19020961

**Published:** 2022-01-15

**Authors:** Federico Roggio, Bruno Trovato, Caterina Ledda, Venerando Rapisarda, Giuseppe Musumeci

**Affiliations:** 1 Department of Biomedical and Biotechnological Sciences, Human, Histology and Movement Science Section, University of Catania, Via S. Sofia 87, 95123 Catania, Italy; federico.roggio@unict.it (F.R.); brunotrovato94@gmail.com (B.T.); 2 Department of Psychology, Educational Science and Human Movement, University of Palermo, Via Giovanni Pascoli 6, 90144 Palermo, Italy; 3 Department of Clinical and Experimental Medicine, Occupational Medicine, University of Catania, Via Santa Sofia 78, 95123 Catania, Italy; caterina.ledda@unict.it (C.L.); vrapisarda@unict.it (V.R.); 4 Research Center on Motor Activities (CRAM), University of Catania, Via S. Sofia n°97, 95123 Catania, Italy; 5 Department of Biology, Sbarro Institute for Cancer Research and Molecular Medicine, College of Science and Technology, Temple University, Philadelphia, PA 19122, USA

**Keywords:** spine osteoarthritis, enduro motorcyclist, prevention, overuse, spine mobility, pain, muscle strength

## Abstract

This case report speculates that the prolonged vibrations from enduro off-road sports are deleterious to the spine. The results of this case report may also aid sports physicians in better understanding this complex and relatively unknown phenomenon. No published data are present in the current literature that demonstrate the correlation between early spine osteoarthritis from enduro motorcycle overuse and the long-term management effects of a non-invasive kinesiological approach to reduce pain and inflammation and improve spine mobility and muscle strength.

## 1. Introduction

Spine osteoarthritis (OA) is widespread, with estimations of its prevalence ranging from 40 to 85% of the entire world population [1]. OA is rarely linked to spinal disease because in the majority of cases, this disease is linked to the peripheral joints (hands and knees) rather than to the spine. The macroscopic evidence reveals disc space narrowing and vertebral osteophyte formation [2]. Although a definite correlation between spine OA and low back pain (LPB) has not been found, several conditions may predispose the spine to degenerate into an osteoarthritic condition. The common causes of low back pain are internal disc disruption, facet joint arthrosis, and sacroiliac joint arthrosis [3], while OA is a disorder of the synovial joints. Both conditions can affect facet and sacroiliac joint arthrosis, causing LBP deriving from OA joint degeneration [4]. As stated by Laplante et al. [4], OA is not the main cause of all spinal joint problems; however, the relative percentage of joint pain due to OA is age-related. LBP is a chronic condition, with nearly 80% of the population undergoing at least one event of LBP in their lifetime [5]. The complicated connection between spine radiographs and LBP has many clinical and research challenges. In individuals with cancer, LBP may stem from conditions that are challenging to identify with radiographs, such as malignant spinal cord compression, which can cause pain, paresthesia, and motor weakness [6]. High-amplitude, low-frequency vibration has been recognized as a risk factor for back pain, intervertebral, and temporomandibular disc degeneration [7,8,9]. Although whole-body vibrations (WBV) have been reported as a valuable treatment for back pain and osteoporosis, other studies suggest that the WBV effects on bones may vary with age, genetics, anatomical location, and application [10]. 

Off-road motorcycling is a hazardous sport with little known about its connection to spine lesions compared to other well-known sports [11]. Off-road motorcyclists are constantly exposed to WBV, which may predispose the body to excessive stress on the musculoskeletal system. The most notorious causes of spinal damage are impact, motorcycle jumping, or the loss of control from high-speed sway [12]. Some lesions, such as vertebral fractures leading to paralysis, permanent handicap, and lethal injuries, have been described in connection with motocross accidents [13]. In this case report, we observed the presence of spine OA in an enduro motorcyclist and how a kinesiological approach determined joint mobility recovery and pain reduction. Recent interest in using exercise therapy and Kinesio taping application showed improvements in treating chronic OA and LBP [14,15]. This case report aims to provide the first documented evidence of early spine osteoarthritis in enduro motorcycle overuse and the long-term management effects of a non-invasive kinesiological approach to reduce pain and inflammation and improve spine mobility and muscle strength.

## 2. Case Report

**Figure 1:**
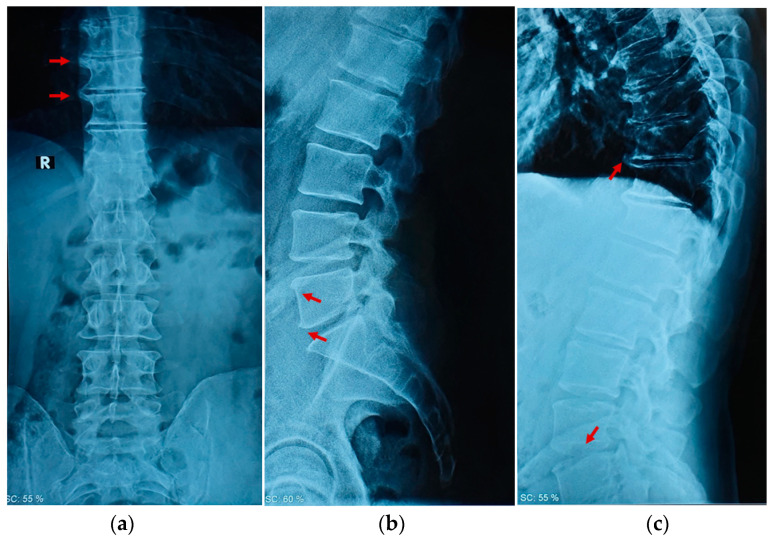
Radiographic examination of the spine: (**a**) represents the coronal plane, and the red arrows indicate the osteoarthritic degeneration at the right side of the vertebrae; (**b**) represents the sagittal plane of the lumbar region, and the red arrows indicate the presence of osteophytes at L5; (**c**) represents the sagittal plane of the thoracic and lumbar region, and the red arrows indicate the osteoarthritic degeneration of both segments.

A 45-year-old off-road motorcyclist—1.78 cm tall, with a weight of 80 kg—experienced acute pain over the thoracolumbar region during a vintage motorcycle race championship in the absence of any accident or critical event to explain the acute pain onset. The patient was clinically evaluated after the race and then forced to suspend the championship due to this condition. Due to the acute pain, an X-ray exam was prescribed. The radiologist’s diagnosis showed the absence of traumatic bone injuries but revealed discrete signs of diffuse osteoarthritis with dorsal interbody bony bridges, moderate osteophytes, and narrowing of disc spaces from T12-L1 and L5-S1; a clinical condition similar to that of an elderly osteoarthritic spine [16], classified as grade 2 on the K&L scale [17], as shown in Figure 1.

**Figure 2:**
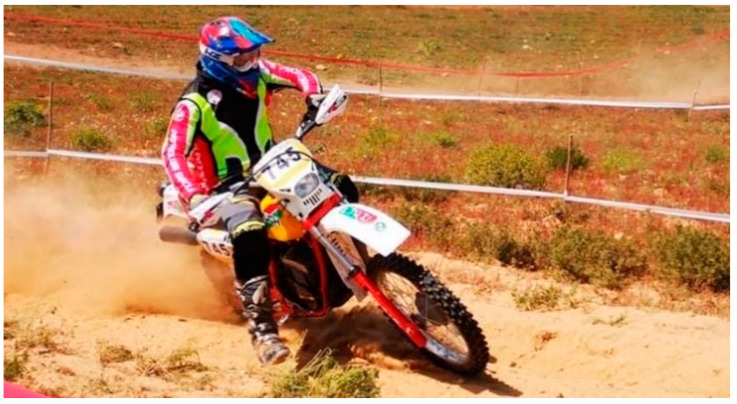
The forty-five-year-old motorcyclist during a race.

The patient, Figure 2, began his motocross career 28 years ago with training approximately two times a week for the entire year. The patient is a labor consultant, an occupation that does not require excessive manual effort. As reported by the patient, the symptoms (inability to fully extend and flex the spine, pain during spine extension and flexion, loss of strength, and difficulty accomplishing natural movements) began mildly one week before the race and reached their peak during the race. The patient reported episodes of low back pain in the previous year but no acute events during that time. The patient’s clinical history of motocross-related injuries included various injuries, such as trauma to the sacral bone eight years prior to the study, a left knee tibial plateau fracture five years prior to the study, and varicose vein surgery in both lower limbs three years prior to the study. The patient’s clinical and family history bore no trace of osteoarthritis, rheumatoid arthritis or microfractures in the spine.

### 2.1. Clinical Examination

This study was approved by the local ethics committee of the Research Center on Motor Activities (CRAM) (Protocol n.: CRAM-017-2020, 16 March 2020) and the University of Catania, in accordance with the Declaration of Helsinki. Informed consent was obtained from the patient. For the clinical evaluation, the following approaches were utilized to collect the data that are most relevant to the case: the Spine 3D (Sensor Medica^®^, Rimini, Italy) was used to evaluate the spinal deformities, if present, through infrared rays. The system generates a 3D model of the spine by calculating specific deformities. It assesses the presence of scoliosis, pelvic obliquity, thoracic kyphosis, lordosis angles, and lateral deviations. BioFet (Fisiotools, Roma, Italy) is a portable electronic dynamometer employed to evaluate the muscle force in the extensor spine muscles. Bobomotion (Fisiotools, Roma, Italy) is a miniature sensor able to analyze a range of motion. Specifically, it has been used to evaluate spine motion in flexion, extension, and inclination. Taping Elastico^®^ (ATS, Arezzo, Italy) is Kinesio taping applied to reduce pain and increase mobility. Muscle force, range of motion, and the Visual Analogue Scale (VAS) score were collected at the beginning of each week for four weeks and then at 3–6 month follow-up intervals.

### 2.2. Physical Examination

**Figure 3:**
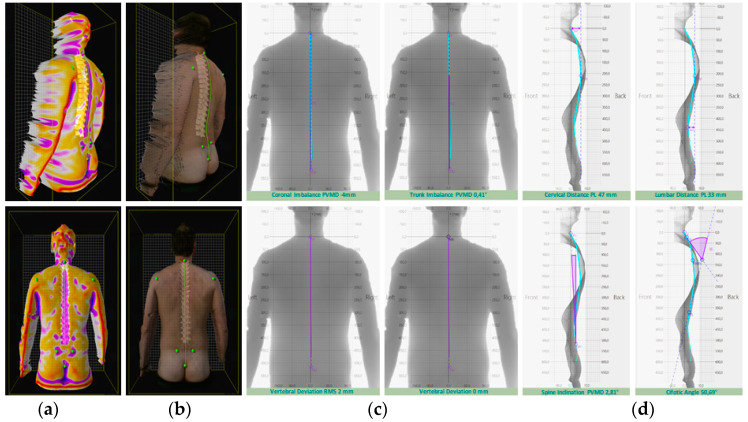
Spine rasterstereography accomplished through the Spine3D system. (**a**) represents the 3D reconstruction of back concavities. (**b**) represents the spine reconstruction over the patient’s spine photo. (**c**) represents the coronal plane measurements. (**d**) represents the sagittal plane measurements.

The patient was lying on the examination table in prone decubitus to evaluate the metameric movement of the lumbar spine [18]. The presence of Maigne’s syndrome [19], i.e., thoracolumbar junction syndrome, was evaluated to discriminate the presence of thoracolumbar joint inflammation and radicular pain. The spinal extension force was evaluated through the dynamometer use. Lying in prone decubitus with his arms by his sides, the patient was required to extend his spine while the clinician exerted posterior resistance through the dynamometer. The spine motion was evaluated with the patient in a sitting position on the examination table with legs not resting on the ground and arms crossed resting on opposite shoulders. The patient was required to perform a lateral inclination to the left and right and then a flexion and extension. A miniature sensor attached to an elastic belt was placed on the chest to evaluate the spine’s range of motion. The spine rasterstereography elaborated the spine’s morphometric analysis to discriminate the presence of scoliosis and the increase or the reduction in the spine’s curves. The patient was required to stand in the upright position for 5 s with the back and the buttocks uncovered to ensure a valid measurement because the pants’ pressure on the buttocks could cause a surface alteration in the back [20]. During the clinical spine examination, the range of motion (ROM) was, respectively: 10° inflection and 5° in extension, 10° in right inclination, and 17° in left inclination. The patient showed signs of mechanical and functional blocks with a range of motion reduction, specifically at the T12-L1 junction and L4-S1 junction, with no radicular pain present. The VAS score was assessed at a value of 8. The spinal extension muscle force was evaluated through the digital dynamometer. The maximal average force exerted was 3.19 kg, and at 1.2 s, muscle contraction sharply stopped. A morphometric spine analysis (Figure 3) was carried out for further investigation. The morphometric spine analysis was conducted to analyze the physio anatomy of the back in its entirety. There were no lateral deviations in the coronal plane and no alterations in the sagittal plane, except for a slight reduction in cervical and lumbar depth. These conditions cannot justify the presence of early spine osteoarthritis.

### 2.3. Therapeutic Intervention

For the first week, the physician prescribed rest and NSAID anti-inflammatory therapy as needed to reduce pain and inflammation, according to the clinical guidelines for managing non-specific low back pain [21]. The physician observed that the functional limitation derived from the osteoarthritic degenerations through radiographic examination of the spine. Then, by the second week, in agreement with the kinesiologist, a non-invasive kinesiological treatment based on antalgic therapy with Kinesio taping application was administered without NSAID anti-inflammatory therapy. The Kinesio taping application was administered twice per week, while the antalgic therapy for gymnasts required administration at home 3 times per week.

### 2.4. Kinesio Taping Application

**Figure 4:**
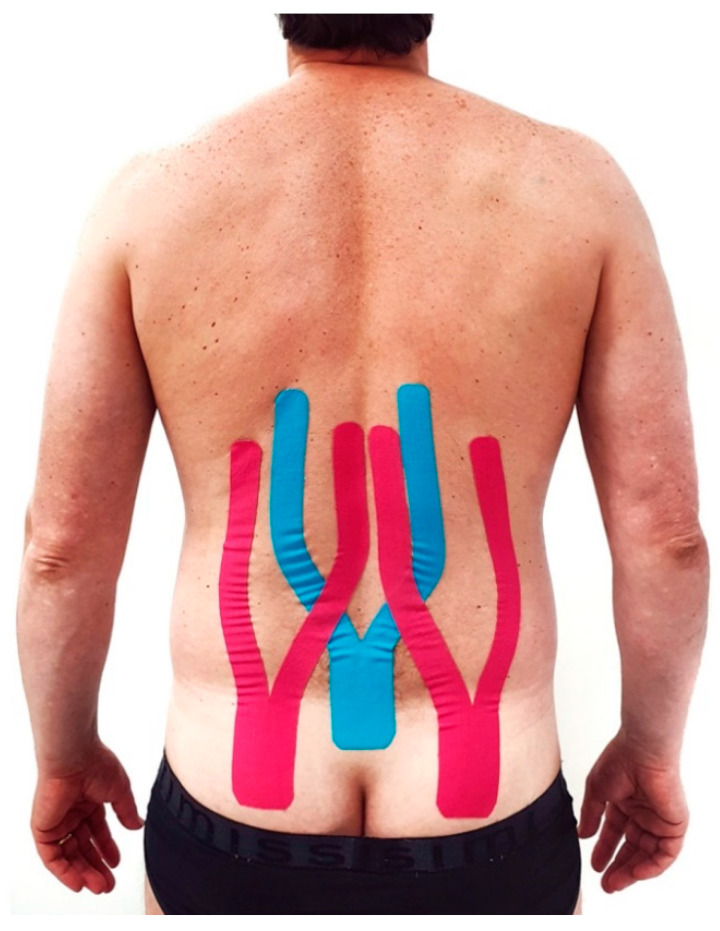
Kinesio taping application in a Y shape. The blue tape is applied over paravertebral chains and the pink tape over the latissimus dorsi and sacrospinalis group of muscles.

Three Kinesio tapes were applied to the patient’s back to reduce pain perception and increase mobility. The tapes were “Y-strips” with a length of approximately 35 cm, an anchor of 8 cm on the buttocks, and applied over the spine up to T10 vertebrae. For the application, the patient was standing in an upright position while the anchor of the blue tapes was placed on the sacrum and the pink tapes on the gluteus maximus. Subsequently, forward bending of the trunk was executed while the two tails of the tapes were applied without stretching because of their stabilizing effect. The blue tails were applied in the direction of the paravertebral chains, and the pink tails were applied in the direction of the latissimus dorsi and sacrospinalis group of muscles, as shown in Figure 4.

### 2.5. Exercise Administration

**Figure 5:**
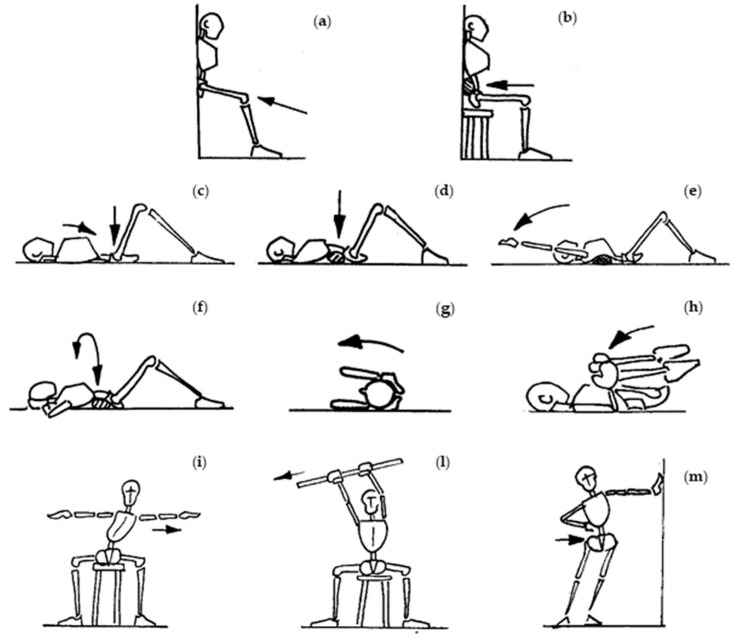
Visual explanation of home-based exercises. Exercises (**a**) and (**b**) aimed to increase the spine’s stability. Exercises (**c**–**h**) aimed to reduce pain perception. Exercises (**i**–**m**) aimed to increase spine mobility.

The following exercises, provided by the ISICO (Italian Spine Scientific Institute) online platform [22,23], were administered to relieve the spine pain and increase mobility (Figure 5). We selected exercises concerning spine mobilization and pain relief, as a number of authors confirmed the high validity of exercise administration in LBP treatment [24,25,26]. During week two, the exercises focused on pain reduction:

1. Upright position with the back leaning against a wall. Bend the knees and push the back against the wall while exhaling (Figure 5a);
2. Sitting position with the back against a wall. Place a rolled-up cloth under the lower back and exert rhythmic pressure while exhaling (Figure 5b);
3. Supine position. Breathe while inflating the belly and chest alternately. With each exhale, press the lower back to the ground (Figure 5c);
4. Supine position. Place a rolled-up cloth in the lumbar area and exert rhythmic pressure while exhaling (Figure 5d);
5. Supine position. Place a cloth under the painful part of the dorsal column and extend the arms while exhaling (Figure 5e);
6. Supine position. Place a cloth in correspondence under the painful area and carry out torsion movements of the trunk while exhaling (Figure 5f);
7. Supine position with legs flexed. Cross roll left and right with hands at the nape of the neck (Figure 5g);
8. Supine position. Flexion of the hips and pull the knees to the chest. Gently swing the trunk left and right (Figure 5h).
  By week three, exercises 6, 7, and 8 were replaced with mobility exercises as follows:
9. Sitting position. Lateral trunk translation in kyphosis with arm extended laterally (Figure 5i);
10. Sitting position with a stick in the hands held high over the head. The lateral inclination of the trunk (Figure 5l);
11. Upright position placed beside a wall with the hand resting and the arm outstretched. With the other hand, push the pelvis towards the wall (Figure 5m).

### 2.6. Changes in Therapeutic Intervention

**Figure 6:**
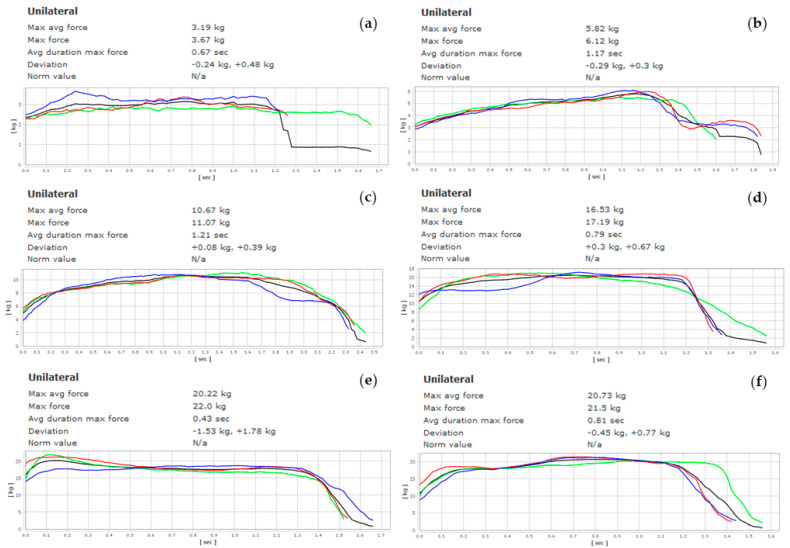
Dynamometer muscle force graph through the weeks: (**a**) week 1, (**b**) week 2, (**c**) week 3, (**d**) week 4, (**e**) 3-month follow-up, (**f**) 6-month follow-up.

The patient reported only transitorily pain relief during week two—the VAS score was 6—and a minimal improvement in the ROM. Based on the clinical outcomes, it was decided to repeat the treatment for a third week, finding a positive improvement in the ROM and a temporary improvement of pain: the VAS score was 4. Moreover, there was no contraindication for repeating the treatment relating to the patient’s general health. Therefore, the treatment was administrated for the fourth week as well. In the last treatment, the patient reported a considerable amount of pain reduction, as confirmed by a VAS score of 2 and a positive improvement in the ROM. Throughout the entire treatment, the patient refrained from driving. In the third and sixth months, the patient repeated the same kinesiological treatment for three weeks without NSAID anti-inflammatory therapy, obtaining positive results in ROM improvement and pain reduction: the VAS score was 1. Spine ROM, VAS, and muscle force (Figure 6) were evaluated each week, as reported in Table 1.

## 3. Discussion

The prevalence of symptomatic spine osteoarthritis in individuals under 50 years of age is very uncommon and challenging to treat [27]. Considering the current literature [28,29,30,31,32] supporting the relationship between vibration and osteoarthritis, our perspective is that over time, the continuous vibrations caused by off-road motorcycle racing can create microtraumas that can lead to spine osteoarthritis. Furthermore, various authors [33,34,35] have analyzed the possible relationship between the WBV experienced by motorcyclists as one of the highest risk factors for low back disorders. For example, as Chen et al. [33] reported, the correct posture while riding is to maintain an upright trunk, as a forward bend in the trunk may predispose motorcyclists to adverse health effects while subjected to WBV. The most frequently injured regions are the extremities (upper and lower limbs) rather than the spine itself [12]. The subject of this study did not report any injury to other body regions aside from the spine. A possible explanation may be the difference in loading between the different spine segments related to driving functions and sitting, especially in stressful conditions such as training and competition. We hypothesize a possible connection between early spine OA diagnosis and the jolting stimulus caused by a motorcycle and rugged terrain for a prolonged period of time. Certainly, early spine OA cannot be univocally attributed to the vibrations caused by enduro off-road sports. However, as highlighted by Tian et al. [36], who investigated the prevalence of degenerative lumbar OA in 3859 Chinese adults, vibrations are reported as one of the higher risk factors associated with spine osteoarthritis among adults (OR: 2.21, 95% CI: 1.51–3.23; *p* < 0.05). These results strengthen the hypothesis that exposure to prolonged vibrations from enduro off-road sports may be deleterious to the spine, especially in this case report where the subject has practiced motorcycling for 28 years and continues to train twice a week. A detailed assumption provided by Patterson et al. [37] points out the possible connection to spine OA indirectly linked to an altered neuromuscular component. WBV could cause muscle inflammation, microtrauma, and nociceptive input to the spinal cord, conditioning the spine biodynamic response under vibrations. Secondly, in silico stress analysis of the lumbar spine, vibrations exposed cancellous bone and the cartilaginous endplate to tissue damage [38]. Vibration treatment applied over musculoskeletal structures could effectively reduce the unfavorable effects of aging on bone, cartilage, muscles, and tendons. However, the vibration parameters, i.e., amplitude, frequency, and magnitude of the oscillations, should be chosen based on the therapeutic need; otherwise, this treatment would have no effect or harmful effects [31,39]. Off-road motorcycling is considered a well-known sport, and because prevention is better than cure, the best support for these athletes could be the implementation of clinical joint examination in the limbs and spine instead of focusing primarily on the cardiorespiratory system [40,41]. Aware of the literature debate around Kinesio taping’s effect on musculoskeletal disorders [42,43,44,45,46,47], we decided to utilize it as a support therapy in the pain management administered through antalgic therapy based on the evidence reported by many authors about the positive effect of Kinesio taping application in conjunction with exercises [46,47,48,49,50,51,52,53,54,55], and its validity in the treatment and prevention of spine injuries [56,57,58,59]. Nevertheless, for low back pain treatment, the kinesiological approach, i.e., exercise administration, is strongly suggested because it effectively generates improvements compared with manual therapy or conservative treatment [60,61,62]. Non-specific LBP, in some cases, may recover without treatment administration; however, in our case, the presence of early spine OA in conjunction with the intense physical effort throughout the enduro championship may have determined the acute LBP, which required treatment to recover spine mobility and strength. Sports physicians are recommended to harvest more data regarding this sport and its related injuries to prevent the development of chronic, disabling diseases from motorcycle overuse and to most effectively address the physical preparation and treatment. Physicians ordinarily suggest pharmacological treatment with or without infiltration to reduce pain and improve the mobility of the spine in patients with OA. In this case, we reported premature spine osteoarthritis in a competitive enduro motorcyclist treated with a conservative, non-invasive, and efficient kinesiological approach. Based on our insights, this scientific contribution could help address this condition and raise questions about a possible negative role of jolting stimulus stress on the spine that might exist among these athletes. 

Certain limitations should be considered, however, when interpreting these findings. First, this is a case study, so we observed that specific condition in one person; second, the results cannot be generalized to the whole osteoarthritic population; third, we cannot unequivocally correlate the early osteoarthritis spine with motorcycling vibrations.

## 4. Conclusions

This is a clinical report in which early spine osteoarthritis from enduro motorcycle overuse is present. The report supports previous findings on early spine osteoarthritis and motorcycling vibrations. This study hypothesizes a possible relationship between long-time vibration exposure and early spine osteoarthritis. Furthermore, we advise a preventive approach and the creation of specific medical examination guidelines for these athletes. Furthermore, to reduce pain and inflammation and improve the spine mobility and muscle strength of these patients/athletes, a non-invasive kinesiological approach should be recommended for the long term. Additional research concerning the Kinesio taping application is required to establish a guideline accepted by the entire scientific community. A specific investigation of the causality between long-time vibration exposure and early spine osteoarthritis is needed.

## Figures and Tables

**Table 1:** Physical evaluation of pain, mobility, and force through the weeks.

	Week 1	Week 2	Week 3	Week 4	3rd month	6th month
VAS scale	8	6	4	2	1	1
ROM flexion	10°	22°	35°	68°	75°	78°
ROM extension	2°	2°	8°	9°	15°	18°
ROM lateral inclination left	17°	20°	36°	55°	63°	67°
ROM lateral inclination right	10°	15°	27°	46°	58°	60°
Muscle force (kg)	3.19	5.82	10.67	16.53	20.22	20.73

VAS, visual analogue scale; ROM, range of motion.

## Data Availability

Data sharing not applicable.
